# *N*-(2-Hy­droxy­benz­yl)adamantan-1-aminium chloride

**DOI:** 10.1107/S1600536811020794

**Published:** 2011-06-11

**Authors:** Tao Rong

**Affiliations:** a Ordered Matter Science Research Center, Southeast University, Nanjing 210096, People’s Republic of China

## Abstract

The asymmetric unit of the title compound, C_17_H_24_NO^+^·Cl^−^, consists of a discrete *N*-(2-hy­droxy­benz­yl)adamantan-1-aminium cation and a Cl^−^ anion. Inter­molecular N—H⋯Cl and O—H⋯Cl hydrogen bonds occurring between the organic cation and the Cl^−^ anion generate a layered structure.

## Related literature

For general background to ferroelectric organic frameworks, see: Ye *et al.* (2006 ▶, 2009 ▶); Fu *et al.* (2007 ▶); Zhao *et al.* (2008 ▶). For phase transitions of ferroelectric materials, see: Zhang *et al.* (2008 ▶).
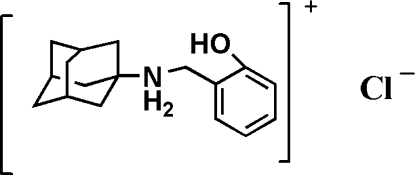


## Experimental

### 

#### Crystal data

C_17_H_24_NO^+^·Cl^−^
*M* *_r_* = 293.82
Monoclinic, 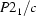

*a* = 12.262 (3) Å
*b* = 10.202 (2) Å
*c* = 12.845 (3) Å
β = 98.43 (3)°
*V* = 1589.5 (6) Å^3^
*Z* = 4
Mo *K*α radiation
μ = 0.24 mm^−1^
*T* = 293 K
0.20 × 0.20 × 0.20 mm

#### Data collection

Rigaku SCXmini diffractometer
Absorption correction: multi-scan (*CrystalClear*; Rigaku, 2005 ▶) *T*_min_ = 0.954, *T*_max_ = 0.954
15541 measured reflections
3632 independent reflections
2249 reflections with *I* > 2σ(*I*)
*R*_int_ = 0.088

#### Refinement

*R*[*F* ^2^ > 2σ(*F* ^2^)] = 0.074
*wR*(*F* ^2^) = 0.217
*S* = 1.06
3632 reflections
204 parameters
H-atom parameters constrained
Δρ_max_ = 0.35 e Å^−3^
Δρ_min_ = −0.48 e Å^−3^

### 

Data collection: *CrystalClear* (Rigaku, 2005 ▶); cell refinement: *CrystalClear*; data reduction: *CrystalClear*; program(s) used to solve structure: *SHELXS97* (Sheldrick, 2008 ▶); program(s) used to refine structure: *SHELXL97* (Sheldrick, 2008 ▶); molecular graphics: *DIAMOND* (Brandenburg & Putz, 2005 ▶); software used to prepare material for publication: *SHELXL97*.

## Supplementary Material

Crystal structure: contains datablock(s) I, global. DOI: 10.1107/S1600536811020794/kp2325sup1.cif

Structure factors: contains datablock(s) I. DOI: 10.1107/S1600536811020794/kp2325Isup2.hkl

Supplementary material file. DOI: 10.1107/S1600536811020794/kp2325Isup3.cml

Additional supplementary materials:  crystallographic information; 3D view; checkCIF report

## Figures and Tables

**Table 1:** Hydrogen-bond geometry (Å, °)

*D*—H⋯*A*	*D*—H	H⋯*A*	*D*⋯*A*	*D*—H⋯*A*
N1—H1*A*⋯Cl1^i^	0.90	2.58	3.260 (2)	133
N1—H1*C*⋯Cl1^ii^	0.90	2.38	3.118 (2)	139
O1—H1*B*⋯Cl1^iii^	0.85	2.27	3.049 (2)	152

Symmetry codes: (i) 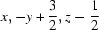; (ii) 
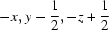
; (iii) 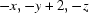.

**Fig. 1.**
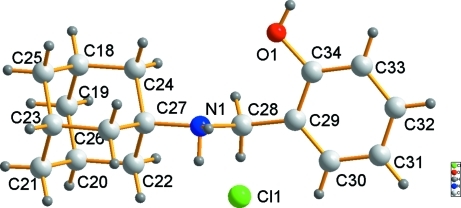
The molecular structure of the title compound with the atomic numbering scheme.

**Fig. 2.**
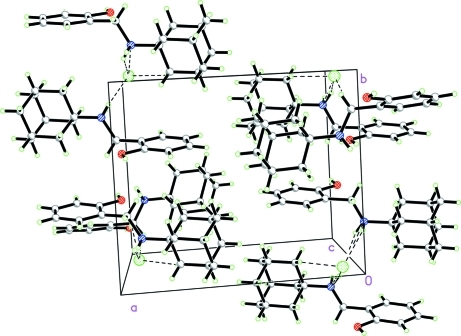
A view of the packing of the title compound, showing hydrogen-bonded helices along the axis b.

## supplementary crystallographic information

### Comment

The study of ferroelectric materials has received much attention. Some materials have predominantly dielectric-ferroelectric performances.The title compound was studied as part of our work to obtain potential ferroelectric phase-change materials (Ye *et al.*, 2006; Fu *et al.*, 2007; Zhao *et al.* 2008; Zhang *et al.*, 2008; Ye *et al.*, 2009). Unluckily, the compound has no dielectric anomalies in the temperature range 93–453 K, suggesting that it might be only a paraelectric. The title compound (Fig. 1), consists of protonated N-(2-hydroxybenzyl)–1–adamantylammonium cation and Cl^-^ anion. In the crystal structure, the N—H···Cl and O—H···Cl hydrogen bonds between the organic cations and the Cl^-^ anions stabilise crystal packing. In the cation, the groups  NH_2_^+^  and OH are proton donors to Cl^-^ forming hydrogen bonds (Table 1, Fig. 2).

### Experimental

KOH (20 mmol)and salicylaldehyde (20 mmol) were added into a solution of amantadine hydrochloride (20 mmol) in ethanol. Then a little of anhydrous magnesium sulfate was added into the mixture, after 6 h the yellow precipitate came out. The yellow solid of amantadine shrink Yang Schiff was obtained by filtration, collection and drying.

NaBH_4_(3.78 g) was added into a solution of amantadine shrink Yang Schiff (25 mmol) in anhydrous methanol(120 mL). After 5 h reaction, a white solid 2-(adamantane-1-aminomethyl)phenol was obtained by reduced pressure distillation, extraction and drying.

A solution of hydrochloric acid (10 mmol) was added to a solution of 2-(adamantane-1-aminomethyl)phenol (10 mmol) in ethanol (20 mL). Crystals suitable for structure determination were grown by slow evaporation of the mixture at room temperature.

### Refinement

Positional parameters of all the H atoms bonded to C atoms were calculated geometrically and were allowed to ride on the C atoms to which they are bonded, with *U*_iso_(H) = 1.2Ueq(C) and *U*_iso_(H) = 1.5Ueq(C) for the methyl group. The other H bonded to O/N atoms were calculated geometrically and were allowed to ride on the O/N atoms with *U*_iso_(H) = 1.2Ueq(N) and *U*_iso_(H) = 1.5Ueq(O).

### Crystal data

**Table:**

C_17_H_24_NO^+^·Cl^−^	*Z* = 4
*M**_r_* = 293.82	*F*(000) = 632
Monoclinic, *P*2_1_/*c*	*D*_x_ = 1.228 Mg m^−^^3^
Hall symbol: -P 2ybc	Mo *K*α radiation, λ = 0.71073 Å
*a* = 12.262 (3) Å	θ = 3.0–27.5°
*b* = 10.202 (2) Å	µ = 0.24 mm^−^^1^
*c* = 12.845 (3) Å	*T* = 293 K
β = 98.43 (3)°	Prism, colourless
*V* = 1589.5 (6) Å^3^	0.20 × 0.20 × 0.20 mm

### Data collection

**Table:**

Rigaku SCXmini diffractometer	3632 independent reflections
Radiation source: fine-focus sealed tube	2249 reflections with *I* > 2σ(*I*)
graphite	*R*_int_ = 0.088
Detector resolution: 13.6612 pixels mm^-1^	θ_max_ = 27.5°, θ_min_ = 3.2°
CCD_Profile_fitting scans	*h* = −15→15
Absorption correction: multi-scan (*CrystalClear*; Rigaku, 2005)	*k* = −13→13
*T*_min_ = 0.954, *T*_max_ = 0.954	*l* = −16→16
15541 measured reflections	

### Refinement

**Table:**

Refinement on *F*^2^	Primary atom site location: structure-invariant direct methods
Least-squares matrix: full	Secondary atom site location: difference Fourier map
*R*[*F*^2^ > 2σ(*F*^2^)] = 0.074	Hydrogen site location: inferred from neighbouring sites
*wR*(*F*^2^) = 0.217	H-atom parameters constrained
*S* = 1.06	*w* = 1/[σ^2^(*F*_o_^2^) + (0.0986*P*)^2^ + 0.3244*P*] where *P* = (*F*_o_^2^ + 2*F*_c_^2^)/3
3632 reflections	(Δ/σ)_max_ < 0.001
204 parameters	Δρ_max_ = 0.35 e Å^−^^3^
0 restraints	Δρ_min_ = −0.48 e Å^−^^3^

### Special details

**Table:**

Refinement. Refinement of *F*^2^ against ALL reflections. The weighted *R*-factor *wR* and goodness of fit *S* are based on *F*^2^, conventional *R*-factors *R* are based on *F*, with *F* set to zero for negative *F*^2^. The threshold expression of *F*^2^ > σ(*F*^2^) is used only for calculating *R*-factors(gt) *etc*. and is not relevant to the choice of reflections for refinement. *R*-factors based on *F*^2^ are statistically about twice as large as those based on *F*, and *R*- factors based on ALL data will be even larger.

### Fractional atomic coordinates and isotropic or equivalent isotropic displacement parameters (Å^2^)

**Table:**

	*x*	*y*	*z*	*U*_iso_*/*U*_eq_	
Cl1	0.04801 (8)	0.97166 (8)	0.33131 (6)	0.0627 (3)	
O1	−0.01490 (19)	0.8664 (2)	−0.12965 (18)	0.0641 (7)	
H1B	−0.0321	0.8850	−0.1947	0.15 (2)*	
N1	0.07621 (18)	0.6830 (2)	0.05578 (17)	0.0391 (5)	
H1C	0.0596	0.6471	0.1155	0.047*	
H1A	0.0400	0.6179	0.0181	0.23 (3)*	
C18	0.3843 (3)	0.7592 (5)	0.0829 (4)	0.0832 (12)	
H18A	0.4245	0.8220	0.0479	0.113 (16)*	
C19	0.4046 (3)	0.7838 (4)	0.1997 (4)	0.0854 (13)	
H19A	0.4819	0.7749	0.2250	0.093 (13)*	
H19B	0.3827	0.8715	0.2141	0.086 (12)*	
C20	0.3412 (3)	0.6858 (4)	0.2566 (3)	0.0709 (11)	
H20A	0.3543	0.7027	0.3309	0.089 (12)*	
C21	0.3796 (3)	0.5474 (4)	0.2345 (3)	0.0752 (11)	
H21A	0.4562	0.5379	0.2621	0.072 (11)*	
H21B	0.3384	0.4840	0.2679	0.100 (15)*	
C22	0.2176 (3)	0.7000 (4)	0.2164 (2)	0.0604 (9)	
H22A	0.1762	0.6394	0.2523	0.086 (13)*	
H22B	0.1938	0.7873	0.2297	0.079 (12)*	
C23	0.3609 (3)	0.5257 (4)	0.1154 (3)	0.0704 (11)	
H23A	0.3858	0.4396	0.0999	0.095 (13)*	
C24	0.2618 (3)	0.7734 (4)	0.0421 (3)	0.0679 (10)	
H24A	0.2377	0.8606	0.0551	0.111 (16)*	
H24B	0.2479	0.7574	−0.0323	0.103 (15)*	
C25	0.4232 (3)	0.6267 (5)	0.0625 (4)	0.0852 (13)	
H25A	0.4139	0.6101	−0.0119	0.130 (19)*	
H25B	0.5004	0.6207	0.0892	0.093 (13)*	
C26	0.2374 (3)	0.5371 (3)	0.0762 (3)	0.0639 (10)	
H26A	0.2226	0.5201	0.0020	0.087 (13)*	
H26B	0.1981	0.4737	0.1116	0.052 (9)*	
C27	0.1979 (2)	0.6736 (3)	0.0991 (2)	0.0409 (6)	
C28	0.0184 (3)	0.8077 (3)	0.0800 (2)	0.0500 (7)	
H28A	0.0613	0.8815	0.0634	0.082 (12)*	
H28B	0.0135	0.8109	0.1539	0.043 (8)*	
C29	−0.0946 (2)	0.8170 (3)	0.0198 (2)	0.0437 (7)	
C30	−0.1871 (3)	0.8027 (3)	0.0692 (3)	0.0625 (9)	
H30A	−0.1780	0.7829	0.1430	0.082 (12)*	
C31	−0.2914 (3)	0.8181 (4)	0.0146 (4)	0.0774 (11)	
H31A	−0.3550	0.8068	0.0493	0.100 (15)*	
C32	−0.3042 (3)	0.8490 (4)	−0.0905 (4)	0.0794 (12)	
H32A	−0.3770	0.8604	−0.1288	0.105 (14)*	
C33	−0.2136 (3)	0.8645 (3)	−0.1424 (3)	0.0653 (10)	
H33A	−0.2225	0.8857	−0.2160	0.101 (14)*	
C34	−0.1099 (3)	0.8494 (3)	−0.0868 (2)	0.0492 (7)	

### Atomic displacement parameters (Å^2^)

**Table:**

	*U*^11^	*U*^22^	*U*^33^	*U*^12^	*U*^13^	*U*^23^
Cl1	0.1004 (7)	0.0557 (5)	0.0339 (4)	0.0196 (4)	0.0159 (4)	0.0021 (3)
O1	0.0775 (16)	0.0677 (16)	0.0505 (14)	0.0146 (12)	0.0211 (11)	0.0142 (11)
N1	0.0488 (14)	0.0375 (12)	0.0313 (11)	0.0016 (10)	0.0073 (10)	0.0006 (9)
C18	0.056 (2)	0.090 (3)	0.107 (3)	−0.010 (2)	0.022 (2)	0.016 (3)
C19	0.055 (2)	0.062 (3)	0.133 (4)	−0.0044 (18)	−0.008 (2)	−0.019 (2)
C20	0.059 (2)	0.101 (3)	0.0477 (19)	0.014 (2)	−0.0074 (16)	−0.0226 (19)
C21	0.057 (2)	0.078 (3)	0.086 (3)	0.0117 (19)	−0.007 (2)	0.014 (2)
C22	0.061 (2)	0.083 (3)	0.0361 (16)	0.0120 (18)	0.0050 (15)	−0.0123 (16)
C23	0.067 (2)	0.055 (2)	0.085 (3)	0.0188 (17)	−0.003 (2)	−0.0219 (19)
C24	0.057 (2)	0.076 (3)	0.073 (3)	−0.0042 (18)	0.0178 (18)	0.017 (2)
C25	0.059 (3)	0.113 (4)	0.087 (3)	0.006 (2)	0.022 (2)	−0.006 (3)
C26	0.068 (2)	0.0471 (19)	0.073 (2)	0.0063 (16)	−0.0012 (18)	−0.0148 (17)
C27	0.0461 (16)	0.0396 (15)	0.0379 (14)	−0.0007 (12)	0.0091 (12)	−0.0024 (11)
C28	0.0632 (19)	0.0418 (17)	0.0436 (16)	0.0065 (14)	0.0024 (14)	−0.0075 (13)
C29	0.0478 (16)	0.0352 (14)	0.0481 (16)	0.0033 (12)	0.0072 (13)	0.0004 (12)
C30	0.067 (2)	0.059 (2)	0.066 (2)	0.0046 (16)	0.0223 (18)	0.0098 (17)
C31	0.053 (2)	0.070 (3)	0.112 (4)	−0.0011 (18)	0.020 (2)	0.005 (2)
C32	0.053 (2)	0.062 (2)	0.114 (4)	0.0038 (17)	−0.016 (2)	−0.006 (2)
C33	0.076 (2)	0.054 (2)	0.060 (2)	0.0130 (17)	−0.0111 (18)	−0.0020 (16)
C34	0.0628 (19)	0.0361 (15)	0.0481 (17)	0.0059 (13)	0.0058 (15)	−0.0010 (12)

### Geometric parameters (Å, °)

**Table:**

O1—C34	1.370 (4)	C23—C26	1.529 (5)
O1—H1B	0.8530	C23—H23A	0.9600
N1—C28	1.511 (3)	C24—C27	1.535 (4)
N1—C27	1.517 (3)	C24—H24A	0.9601
N1—H1C	0.9000	C24—H24B	0.9600
N1—H1A	0.9001	C25—H25A	0.9600
C18—C25	1.470 (6)	C25—H25B	0.9600
C18—C19	1.505 (6)	C26—C27	1.517 (4)
C18—C24	1.524 (5)	C26—H26A	0.9601
C18—H18A	0.9599	C26—H26B	0.9601
C19—C20	1.518 (6)	C28—C29	1.488 (4)
C19—H19A	0.9600	C28—H28A	0.9601
C19—H19B	0.9599	C28—H28B	0.9600
C20—C21	1.528 (5)	C29—C30	1.386 (4)
C20—C22	1.534 (4)	C29—C34	1.394 (4)
C20—H20A	0.9600	C30—C31	1.375 (5)
C21—C23	1.529 (6)	C30—H30A	0.9600
C21—H21A	0.9600	C31—C32	1.373 (6)
C21—H21B	0.9599	C31—H31A	0.9601
C22—C27	1.515 (4)	C32—C33	1.386 (6)
C22—H22A	0.9599	C32—H32A	0.9602
C22—H22B	0.9600	C33—C34	1.373 (5)
C23—C25	1.504 (6)	C33—H33A	0.9598
			
C34—O1—H1B	108.6	C18—C24—H24B	110.4
C28—N1—C27	116.3 (2)	C27—C24—H24B	109.5
C28—N1—H1C	89.7	H24A—C24—H24B	108.4
C27—N1—H1C	89.7	C18—C25—C23	110.5 (3)
C28—N1—H1A	121.6	C18—C25—H25A	110.2
C27—N1—H1A	122.1	C23—C25—H25A	109.6
H1C—N1—H1A	90.2	C18—C25—H25B	108.9
C25—C18—C19	108.7 (4)	C23—C25—H25B	109.5
C25—C18—C24	110.8 (4)	H25A—C25—H25B	108.1
C19—C18—C24	109.8 (3)	C27—C26—C23	109.6 (3)
C25—C18—H18A	109.1	C27—C26—H26A	109.8
C19—C18—H18A	109.5	C23—C26—H26A	110.3
C24—C18—H18A	108.9	C27—C26—H26B	109.2
C18—C19—C20	110.4 (3)	C23—C26—H26B	109.5
C18—C19—H19A	109.5	H26A—C26—H26B	108.3
C20—C19—H19A	109.0	C22—C27—N1	111.0 (2)
C18—C19—H19B	109.7	C22—C27—C26	110.3 (3)
C20—C19—H19B	110.1	N1—C27—C26	108.2 (2)
H19A—C19—H19B	108.3	C22—C27—C24	109.7 (3)
C19—C20—C21	109.0 (3)	N1—C27—C24	109.1 (2)
C19—C20—C22	109.1 (3)	C26—C27—C24	108.5 (3)
C21—C20—C22	109.9 (3)	C29—C28—N1	112.1 (2)
C19—C20—H20A	109.7	C29—C28—H28A	109.4
C21—C20—H20A	109.9	N1—C28—H28A	109.0
C22—C20—H20A	109.3	C29—C28—H28B	108.8
C20—C21—C23	108.4 (3)	N1—C28—H28B	109.4
C20—C21—H21A	109.6	H28A—C28—H28B	108.0
C23—C21—H21A	110.5	C30—C29—C34	118.3 (3)
C20—C21—H21B	110.1	C30—C29—C28	121.2 (3)
C23—C21—H21B	109.8	C34—C29—C28	120.4 (3)
H21A—C21—H21B	108.5	C31—C30—C29	121.1 (3)
C27—C22—C20	108.9 (3)	C31—C30—H30A	119.5
C27—C22—H22A	110.2	C29—C30—H30A	119.4
C20—C22—H22A	110.1	C32—C31—C30	119.4 (4)
C27—C22—H22B	109.5	C32—C31—H31A	120.1
C20—C22—H22B	109.8	C30—C31—H31A	120.5
H22A—C22—H22B	108.4	C31—C32—C33	121.0 (3)
C25—C23—C21	110.0 (3)	C31—C32—H32A	119.6
C25—C23—C26	110.1 (3)	C33—C32—H32A	119.4
C21—C23—C26	108.3 (3)	C34—C33—C32	119.0 (4)
C25—C23—H23A	109.6	C34—C33—H33A	120.0
C21—C23—H23A	109.6	C32—C33—H33A	121.1
C26—C23—H23A	109.3	O1—C34—C33	123.7 (3)
C18—C24—C27	108.6 (3)	O1—C34—C29	115.1 (3)
C18—C24—H24A	110.0	C33—C34—C29	121.2 (3)
C27—C24—H24A	109.9		

### Hydrogen-bond geometry (Å, °)

**Table:**

*D*—H···*A*	*D*—H	H···*A*	*D*···*A*	*D*—H···*A*
N1—H1A···Cl1^i^	0.90	2.58	3.260 (2)	133.
N1—H1C···Cl1^ii^	0.90	2.38	3.118 (2)	139.
O1—H1B···Cl1^iii^	0.85	2.27	3.049 (2)	152.

Symmetry codes: (i) *x*, −*y*+3/2, *z*−1/2; (ii) −*x*, *y*−1/2, −*z*+1/2; (iii) −*x*, −*y*+2, −*z*.
